# Surgical Aortic Valve Replacement—Age-Dependent Choice of Prosthesis Type

**DOI:** 10.3390/jcm10235554

**Published:** 2021-11-26

**Authors:** Keti Vitanova, Felix Wirth, Johannes Boehm, Melchior Burri, Rüdiger Lange, Markus Krane

**Affiliations:** 1 Department of Cardiovascular Surgery, German Heart Center Munich, Technische Universität München, 80636 Munich, Germany; wirth@dhm.mhn.de (F.W.); johannes.boehm@dhm.mhn.de (J.B.); burri@dhm.mhn.de (M.B.); lange@dhm.mhn.de (R.L.); krane@dhm.mhn.de (M.K.); 2 Insure (Institute of Translational Cardiac Surgery), Department of Cardiovascular Surgery, German Heart Center Munich, Technische Universität München, 80636 Munich, Germany; 3 DZHK (German Center for Cardiovascular Research), Partner Site Munich Heart Alliance, 80636 Munich, Germany; 4 Division of Cardiac Surgery, Department of Surgery, Yale University School of Medicine, New Haven, CT 06511, USA

**Keywords:** SAVR, bioprosthesis, mechanical prosthesis, aortic valve replacement

## Abstract

Background: Recently, the use of surgically implanted aortic bioprostheses has been favoured in younger patients. We aimed to analyse the long-term survival and postoperative MACCE (Major Adverse Cardiovascular and Cerebral Event) rates in patients after isolated aortic valve replacement. Methods: We conducted a single-centre observational retrospective study, including all consecutive patients with isolated aortic valve replacement. 1:1 propensity score matching of the preoperative baseline characteristics was performed. Results: A total of 2172 patients were enrolled in the study. After propensity score matching the study included 428 patients: 214 biological vs. 214 mechanical prostheses, divided into two subgroups: group A < 60 years and group B > 60 years. The mean follow-up time was 7.6 ± 3.9 years. Estimated survival was 97 ± 1.9% and 89 ± 3.4% at 10 years for biological and mechanical prosthesis, respectively in group A (*p* = 0.06). In group B the survival at 10 years was 79.1 ± 5.8% and 69.8 ± 4.4% for biological and mechanical prosthesis, respectively (*p* = 0.83). In group A, patients with a bioprosthesis exhibited a tendency for higher cumulative incidence MACCE rates compared to patients with a mechanical prosthesis, *p* = 0.83 (bio 7.3 ± 5.3% vs. mech 4.6 ± 2.2% at 10 years). In group B, patients with a mechanical prosthesis showed a tendency for higher cumulative incidence MACCE rates compared to patients with bioprosthesis, *p* = 0.86 (bio 4.3 ± 3.1% vs. mech 9.1 ± 3.1% at 10 years). Conclusions: Long-term survival after surgical aortic valve replacement is similar in patients with a biological and mechanical prosthesis, independent of the patients’ age. Moreover, younger patients (<60 years) with bioprosthesis showed a survival benefit, compared to patients with mechanical prosthesis in this age group.

## 1. Introduction

Aortic valve replacement is the standard procedure to treat severe aortic stenosis. Two types of prosthesis can be surgically implanted: mechanical and bioprosthetic valves, and each type has associated risks and benefits. Biologic prosthetic valves are associated with a higher risk of reoperation than mechanical valves because of structural valve deterioration, but mechanical valves typically necessitate lifelong anticoagulation, which increases the risk of haemorrhage and thromboembolism [1,2,3].

The current ESC-EACTS Guidelines state that mechanical prostheses should be considered in patients aged <60 years for prostheses in the aortic position and aged <65 years for prostheses in the mitral position [4]. These class IIa statements are underlined with another recommendation for the use of a mechanical prosthesis in patients at risk of accelerated structural valve deterioration (young age < 40 years, hyperparathyroidism and hemodialysis [5]. Given the fact, the available prostheses have been nowadays superseded by more durable and less thrombogenic models [6,7,8]. Moreover, there is an ongoing growing tendency worldwide in choosing a bioprosthesis by young patients.

## 2. Methods

### 2.1. Study Design

This study presents a large single-centre, retrospective analysis of all consecutive patients, who underwent isolated surgical aortic valve replacement (SAVR) in the German Heart Centre Munich between 2001 and 2015. Patients‘ data were identified from our internal clinical database. All medical reports, including operative protocols, in-hospital and outpatient notes were reviewed. The study was approved by the Institutional Review Board of the Technical University of Munich (Number: 55/21 S). The approval included a waiver of informed patient consent.

Exclusion criteria were prior replacement of any valve and concomitant other valve replacement or repair, coronary artery bypass surgery and thoracic aortic surgery. Selection criteria resulted in a total sample of 2172 patients, of whom 1845 received a biological and 327 a mechanical prosthesis. The initial choice of the valve prosthesis was made according to the patient’s decision. There is a growing tendency among patients to choose a biological prosthesis also at younger age despite the recommendations of the ESC/EACTS Guidelines for management of valvular heart disease from 2017 and 2021 [5,9], where mechanical prostheses are recommended for patients younger than 60 years. 

Therefore, we divided all patients from the identified cohort into two groups according to patients‘ age at the time of surgery: group A—patients younger than 60 years, and group B—patients older than 60 years. 

### 2.2. Endpoints

End-points of the study were all-cause mortality and MACCE (major adverse cardiovascular and cerebral events). MACCE were defined as combined end-point for 30-days all-cause death; stroke; any cause AV-prostheses reoperation; or any major bleeding event, in accordance with the Valve Academic Research Consortium 2 criteria [10].

All patients with mechanical valves were discharged under lifelong anticoagulation therapy (Phenprocoumon) to achieve a targeted international normalized ratio (INR) between 2 and 3. All patients with biological valves were given at discharge Phenprocoumon for at least 3 months, or a lifelong regimen if there were other indications for anticoagulation.

### 2.3. Definitions

Stroke—episode of focal or global neurological deficit (≥24 h; or <24 h if available neuroimaging documents; or the neurological deficit results in death) with at least one of the following: change in the level of consciousness, hemiplegia, hemiparesis, numbness, or sensory loss affecting one side of the body, dysphasia or aphasia, hemianopia, amaurosis fugax, or other neurological signs or symptoms consistent with stroke.

Bleeding—defined as life-threatening, major or minor bleeding according to the BARC criteria [10].

### 2.4. Statistical Analysis

All statistical analyses were performed using Statistical Package for the Social Sciences (SPSS), version 24.0 for Windows (IBM, Ehningen, Germany), R-Studio for Statistical Computing and Data Science (version i386 3.2.0, RStudio, Boston, MA, USA) and NCSS Data Analysis Software (version 2020, NCSS LLC, Kaysville, UT, USA). Categorical variables are presented as absolute numbers and percentages. A Chi-square test (Fisher’s correction test) was used for categorical data between groups. Continuous variables are expressed as means ± standard deviations or medians with minimum and maximum ranges, as appropriate. An independent sample *t*-test was used to compare groups with normally distributed variables and the Mann–Whitney test was used for variables that were not normally distributed. To adjust for potential differences between groups in terms of initial preoperative characteristics or selection bias, analyses of both raw data and of subsequent paired propensity scores were conducted. The 1:1 nearest neighbour matching comparison method without replacement was applied. Kaplan–Meier survival curves were computed after the propensity score matching. Differences in the end-points were evaluated using the log-rank Mantel–Cox test and the Cumulative Incidence Analysis following the Grey’s test including hazard ratio (HR) with 95% confidence intervals (CIs).

## 3. Results

### 3.1. Patients‘ Characteristics

**Figure 1:**
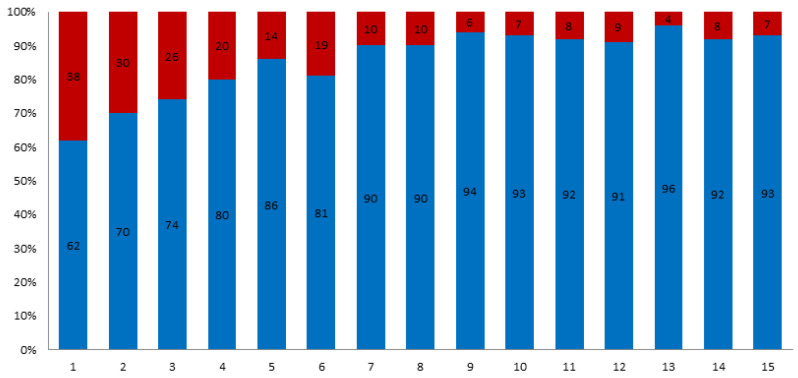
Distribution of biological prosthesis (blue) vs. mechanical (red) for isolated SAVR between 2001 and 2015.

A total of 2172 patients who had undergone isolated primary AVR were initially identified. Follow-up was completed in 81% of the cohort. Overall mean follow-up time was 7.6 ± 3.9 years. The proportion of patients who underwent bioprosthetic AVR increased from 62% in 2001 to 93% in 2015 (Figure 1). Propensity score matching resulted in 428 patients with 214 patient pairs. Follow-up was completed in 83% of the matched cohort. In the study cohort, those patients who received a bioprosthesis were older (69 ± 10 years vs. 54 ± 13, *p* < 0.01) and more likely to have hyperlipoproteinemia (38% vs. 2.7%), arterial hypertension (76% vs. 56%), diabetes mellitus (18% vs. 10%) and history of malignant tumour (8% vs. 2.7%). After propensity score matching, age and all baseline comorbidities were balanced between the 2 groups (Table 1). After propensity score matching the baseline characteristics of the patients younger than 60 years were similar to those of patients older than 60 years (Table 2). After discharge 80% of the patients with biological, and 97.8% of the patients with mechanical prostheses used home-INR monitoring. The INR—values of the other patients were controlled due to the referring cardiologists or family doctors. 

### 3.2. Mortality

**Figure 2:**
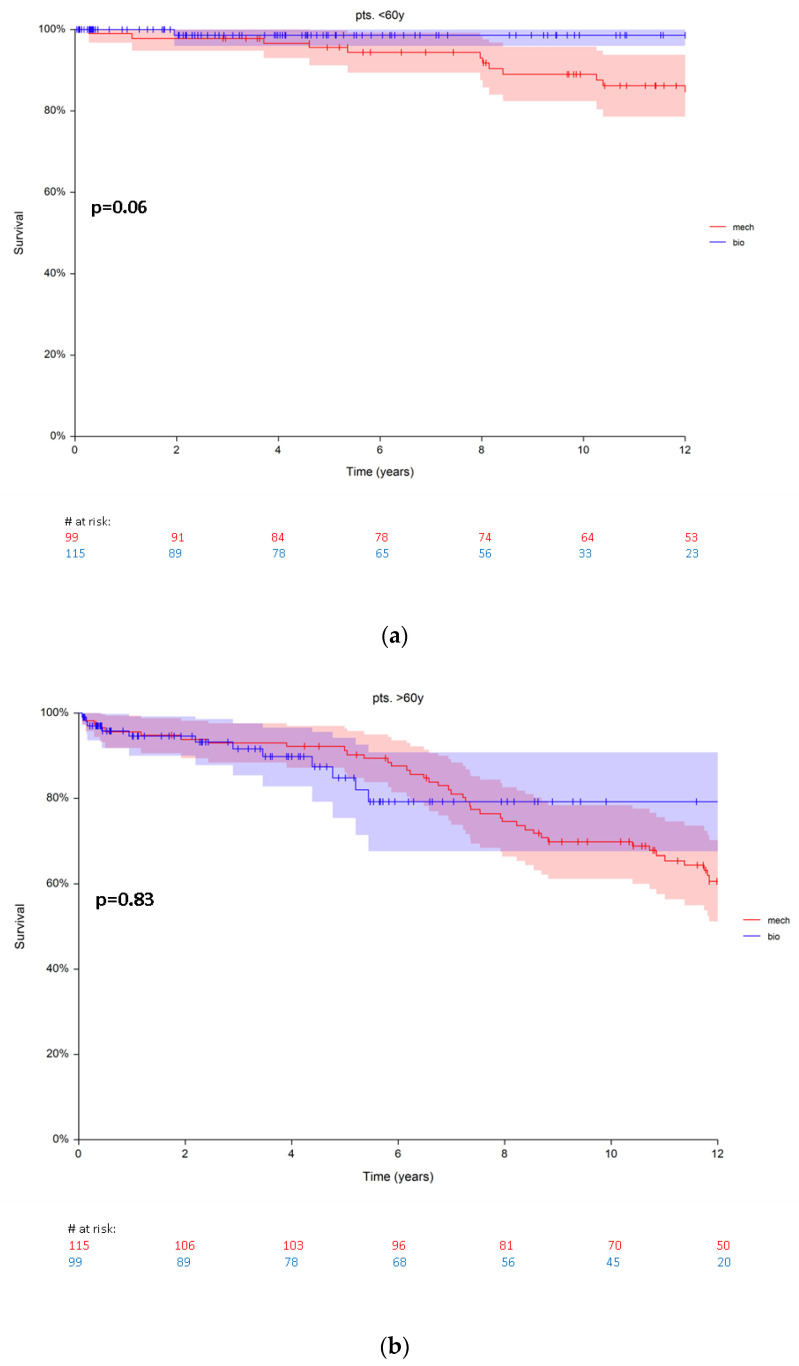
(**a**) Survival in patients < 60 years (group A) with biological vs. mechanical prosthesis. (**b**) Survival in patients > 60 years (group B) with biological vs. mechanical prosthesis. Red numbers are presenting the patients with mechanical prosthesis, blue numbers are presenting the patients with bioprosthesis.

There were 15 (7%) deaths in group A and 60 (28%) deaths in group B during a maximum follow-up of 16.9 years. For long-term survival in group A (pts. < 60 years) a borderline p-value (*p* = 0.06) was observed in favour of bioprosthetic aortic valves. Estimated patients‘ survival was 98.6 ± 1.3 and 95.5 ± 2% at 5 years; 97 ± 1.9% and 89 ± 3.4% at 10 years for biological and mechanical prosthesis, respectively (Figure 2a). The hazard ratio for death was 1.6 (95% CI 1.6–16.4) in group A.

No significant difference in the survival rates was observed comparing patients with biological and mechanical prostheses (*p* = 0.83) in group B (pts. > 60 years). Estimated survival rates were 84.8 ± 4.7% and 92.1 ± 2.6% at 5 years for biological and mechanical prosthesis, respectively. At 10 years the survival rates were 79.1 ± 5.8% and 69.8 ± 4.4% for biological and mechanical prosthesis, respectively (Figure 2b). The hazard ratio for death was 1.0 (0.57–1.98) in group B.

### 3.3. Major Adverse Cardiovascular and Cerebral Events (MACCE)

**Figure 3:**
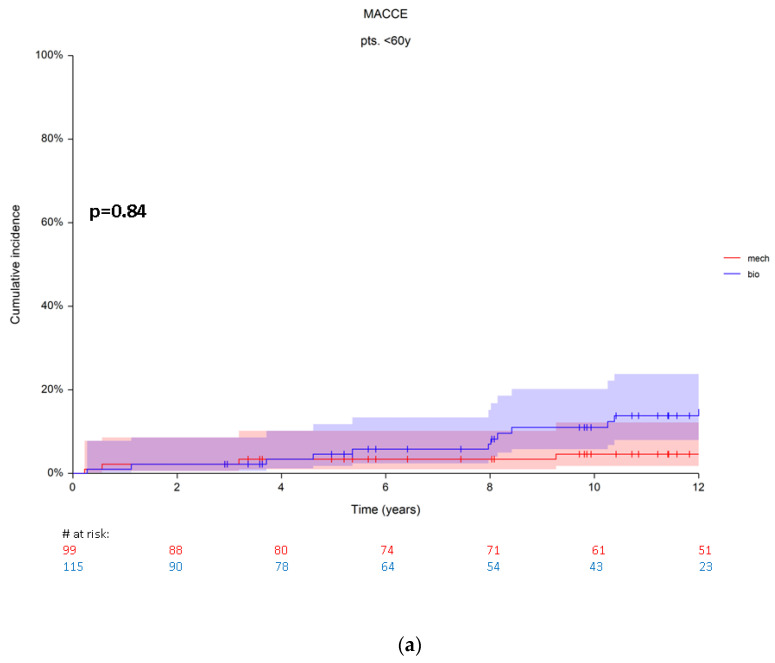

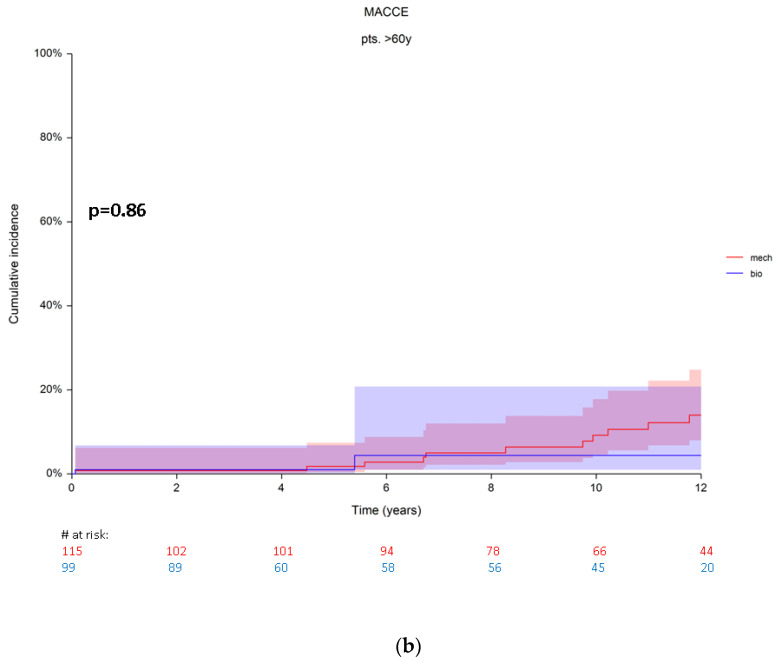
(**a**) Cumulative incidence rates for MACCE in patients from group A. (**b**) Cumulative incidence rates for MACCE in patients from group B. Red numbers are presenting the patients with mechanical prosthesis, blue numbers are presenting the patients with bioprosthesis.

After propensity score matching, 15 patients (7%, *n* = 12 bioprosthetic vs. *n* = 3 mechanical prosthesis) were found with MACCE in group A. At 5 years the cumulative incidence rates were 3.3 ± 1.8 (95% CI 1.01–10) and 2.3 ± 1.9% (95% CI 0.3–15) for bioprosthetic and mechanical replacement, respectively. At 10 years the cumulative incidence rates were also higher in patients with a bioprosthesis (7.3 ± 5.3, 95%CI 1.7–15.9) compared to the cumulative incidence rates (4.6 ± 2.2, 95% CI 1.7–12) in patients with a mechanical prosthesis (Figure 3a). Using Grey’s test, which compares the subdistribution hazards of both, this difference was not significant (*p* = 0.84).

A total of 23 patients (10.7%, *n* = 3 bioprosthesis, vs. *n* = 20 mechanical prosthesis) with MACCE were found in group B. The cumulative incidence rates were at 5 years 0.9 ± 0.1% (95% CI 0.1–6.8) and 1.8 ± 1.3 (95% CI 0.4–7.3) for bioprostheses and mechanical prostheses, respectively. At 10 years patients with a mechanical prosthesis showed a tendency for higher cumulative MACCE incidence rates with 9.1 ± 3.1% (95% CI 4.6–17), compared to 4.3 ± 3.1 (95% CI 4.6–17) for bioprosthesis. The difference revealed no statistical significance (Grey’s *p* = 0.86) also in this patient group (> 60 years) because of the similar subdistribution of their hazards (Figure 3b).

Single adverse events (reoperation, stroke, major bleeding) are reported in the Supplementary Materials Figures S1 and S2.

## 4. Discussion

### 4.1. Growing Trend to Choose Bioprosthetic Replacement

In prosthetic valve selection, mechanical valve-associated thromboembolism and bleeding risk is generally weighed against the risk of SVD and subsequent reintervention associated with biological valve substitutes. In young patients, a mechanical valve is often recommended due to the limited durability of biological valves. However, the durability of contemporary bioprosthetic devices is improving, as well as the outcomes in reoperative aortic valve surgery. These developments together with the prospect of later transcatheter valve-in-valve implantation in failing bioprostheses have led to an increased use in younger patients [11,12]. 

The absence of a significant survival benefit associated with one prosthesis type over another, focuses decision making on lifestyle considerations, including the burden of anticoagulation medication and monitoring, and the relative risks of major morbidity—primarily stroke, reoperation, and major bleeding events. In terms of long-term survival, reported results are more contradictory. Nowadays, approximately 80% of implanted prostheses in Europe and the US are biological valves and this trend is likely to increase [13]. In 2017, a consensus document by the American College of Cardiology and American Heart Association proposed to lower the age limit from 60 to 50 years for the use of biological prosthesis [4]. These recommendations are supported by recent observational studies that have not shown any difference in survival in patients between 50 and 70 years of age with mechanical versus biological aortic valves [2,4]. However, Goldstone et al. found a survival benefit for patients with mechanical prostheses in patients < 55 years. Other researchers have also reported better long-term survival for patients with mechanical prostheses who are in this age range [3,14]. In 2016, Zhao and colleagues published the only meta-analysis on this topic. Despite performing the random effect inverse variance method, which can hide heterogeneity, their pooled analysis on the basis of aggregate data showed high levels of inconsistency (*p* = 0.04, I^2^ = 58%). However, they considered their pooled analysis valid and concluded that long-term survival was similar between patients with mechanical and biological prosthesis [15]. Although some researchers have reported a survival benefit for patients with mechanical prosthesis [3,14], others did not find these differences [2]. We found no difference regarding the long-term survival between patients who received a bioprosthesis compared to a mechanical prosthesis in the group > 60 years old. Interestingly, our analysis showed in patients younger than 60 years an improved long-term survival for biological valve replacement compared to mechanical valve replacement (97 ± 1.9% and 89 ± 3.4% at 10 years) although the difference showed only a borderline significance (*p* = 0.06). We made subanalyses comparing other age groups (<50, <55) and did not find any significant difference regarding mortality. Similarly, recent observational studies have shown at least equivalent mortality, regardless of valve type or position, among patients 50 to 69 years of age [2,16]. Hence our data and in part published data strongly support the escalating use of bioprosthetic valves in younger patients [17]. However, the choice of prosthesis for valve replacement should still be determined in a patient centered approach by balancing the risks of anticoagulation and reoperation.

### 4.2. Pros and Cons of Both Options Regarding MACCE

Diaz et al. confirmed in their meta-analysis that patients with mechanical prostheses have lower rates of reoperation but higher rates of major bleeding events than patients with biological valves [18]. In contrast, stroke rates are similar between both groups. We did not find any significant difference in the cumulative MACCE rates between patients with biological and mechanical prostheses in both groups using a propensity matched approach. Although there was a tendency to higher MACCE rates for patients with bioprosthesis in group A (bio 7.3% vs. mech 4.6% at 10 years) and for those with mechanical prosthesis in group B (bio 4.3% vs. mech 9.1% at 10 years), the difference was not statistically significant. Hence, non-inferiority of the MACCE rates is important since MACCE rates seem to have an influence on the long-term survival. For example, long-term survival is likely to be determined according to anticoagulation-related complications and the risk of reoperation. Therefore, any setting that modifies the proportion of these late complications might have an influence on survival. Therefore, high variability of the International Normalized Ratio (INR) is a strong independent predictor of reduced survival after valve replacement and there is now evidence that INR self-management reduces INR variability and clinical events [9]. Likewise, perioperative mortality of redo surgery or transcatheter valve-in-valve procedures will have an effect on the long-term survival of middle-aged patients with a bioprosthesis [18]. It has been previously reported that reoperation is more common among recipients of a biologic prosthesis compared to mechanical valves [2,3,8,16,19,20]. We observed these findings only in patients younger than 60 years. On the contrary, in our study population of patients older than 60 years, those with mechanical valves required more often reoperation of the aortic valve prosthesis compared to patients with bioprosthesis (bio 0% vs. mech 3.8 ± 1.8%). In the literature, approximately 8–15% of patients with mechanical prostheses require reintervention during their lifetime, mostly due to non-structural valve deterioration (pannus formation, paravalvular leakage, etc.), valve thrombosis or prosthetic valve endocarditis. Although the risk of reintervention after mechanical AVR is low, it is certainly not absent and should be taken into consideration in the process of prosthetic valve selection. This also applies to the risk of thromboembolism and bleeding after surgical AVR with biological alternatives [21]. Our data confirm this statement, as we could not find any difference regarding stroke and bleeding rates between biological and mechanical prosthesis in both patient groups. Taken together, the benefits and limitations of each valve option have substantial implications for life-, career and even pregnancy planning in these patients. Therefore, the conveyance of patient-tailored evidence-based risks and benefits of both mechanical and biological valve options in a shared decision-making process is of great importance [4,5]. 

### 4.3. Options and Future Prospects

Findings from Brown et al. and Glaser et al. contradict the last recommendations of the American Heart Association and American College of Cardiology, as both reported on survival benefits of mechanical valves for patients between 50 and 70 years [3,14]. The differences in survival in both studies are small and become evident during the first years and slightly increase as time goes by. Up to date, the recommendations of valve prosthesis choice in young patients are still confusing and contradictory. A cohort study involving 1001 matched pairs of patients 50 to 69 years of age who underwent isolated aortic-valve replacement showed no difference in mortality between prosthesis types [2]. On the contrary, a subgroup analysis involving 287 matched pairs of patients in a similar study conducted in Sweden showed that mechanical prostheses were associated with substantially lower mortality than biologic prostheses among patients 50 to 59 years old [3]. Brown et al. reported a 10-year survival benefit in 250 matched pairs aged 50 to 70 years favouring mechanical prostheses [14]. This may reflect a systematic treatment bias arising from the tendency to implant mechanical prosthetic valves in healthier patients with better life expectancy. Even after propensity matching, the patients who received bioprosthetic valves in their study were older, had more comorbidities (lung disease and peripheral vascular disease) and had a significantly higher 30-day mortality. McClure et al. compared 310 propensity matched pairs younger than 65 years that were more closely matched (30-day mortality was the same) [22]. In their single-centre analysis, no significant survival difference was found up to 18 years after surgery. In our study population, we could not identify any benefit of the mechanical prosthesis over the bioprosthesis in both age groups. Furthermore, patients > 60 years receiving a mechanical prosthesis had more reoperations, strokes and bleeding events compared to patients with bioprostheses. Additionally, patients < 60 years with bioprosthetic replacement revealed better long-term survival compared to those with a mechanical prosthesis. 

Nowadays, the search for alternative methods to overcome the drawbacks of conventional prosthetic valve replacement is increasing. Aortic valve neocuspidization (AV Neo) for trileaflet aortic valve reconstruction using autologous pericardium (Ozaki procedure) is one very promising method with low mid-term reoperation rates and excellent hemodynamic performance [23]. Nevertheless, long-term results are still pending. Additionally, the Ross procedure represents another valuable option in younger patients to avoid permanent anticoagulation, which may provide sufficient long-term survival, excellent hemodynamic performance and a low risk of endocarditis in selected patients when performed in centres of high expertise [24]. Moreover, with the expansion of the transcatheter development, more attention should be paid in the nearest future on standardized protocols for bioprosthetic valve fracturing during the valve-in-valve procedure. 

## 5. Limitations

This study is limited by its retrospective and non-randomized single-centre design. Additionally, data about the reason for redo was incomplete at the follow-up time. The available echocardiographic reports have been performed in a variety of clinical settings. Furthermore, the reason for death was not specified in our cohort, mortality rates were analysed as “all-cause” death.

## 6. Conclusions

This study showed a comparable survival in patients (<60 years) undergoing biological AVR compared to mechanical prosthesis in our study population. Furthermore, mechanical prosthesis showed no benefit over bioprosthetic replacement regarding cumulative MACCE rates in patients < 60 years. Patients > 60 years, receiving a mechanical prosthesis showed a tendency towards higher cumulative valve reoperation, strokes and bleeding rates.

## Figures and Tables

**Table 1:** Baseline characteristics of all patients before and after propensity score matching.

Before Propensity Score Matching				
	Bioprosthesis *n* = 1845	Mechnical Prosthesis *n* = 327	*p*	Standardised Mean Difference
Age, years	69 ± 10	54 ± 13	<0.01	−2.04
Sex, men	1064 (57)	229 (70)	<0.01	−4.18
Weight, kg	79 ± 15	78 ± 10	0.06	−1.34
Height, cm	179 ± 16	177 ± 14	0.15	−1.40
Chronic obstructive pulmonary disease, *n* (%)	188 (10)	25 (7.6)	0.19	−1.43
Peripheral vascular disease, *n* (%)	66 (3.5)	8 (2.4)	0.29	−1.04
Hyperlipoproteinemia, *n* (%)	702 (38)	88 (2.7)	<0.01	−3.86
Arterial hypertension, *n* (%)	1403 (76)	185 (56)	<0.01	−7.33
Pulmonary hypertension, *n* (%)	249 (13.5)	42 (12.8)	0.74	−3.21
Diabetes mellitus, *n* (%)	336 (18)	33 (10)	<0.01	−3.60
Stroke, *n* (%)	66 (3.5)	8 (2.4)	0.40	−1.04
Cancer, *n* (%)	151 (8)	9 (2.7)	0.001	−3.46
After Propensity Score Matching				
	Bioprosthesis *n* = 214	Mechnical prosthesis *n* = 214	*p*	Standardised Mean Difference
Age, years	60 ± 10	60 ± 9	0.14	−3.45
Sex, men	149 (69.6)	140 (65)	0.40	1.92
Weight, kg	81 ± 16	80 ± 14	0.86	−2.90
Height, cm	180 ± 15	179 ± 16	0.98	−1.94
Chronic obstructive pulmonary disease, *n* (%)	8 (3.7)	16 (7.4)	0.14	1.67
Peripheral vascular disease, *n* (%)	1 (0.5)	5 (2.3)	0.21	1.64
Hyperlipoproteinemia, *n* (%)	87 (40)	66 (30)	0.04	−2.11
Arterial hypertension, *n* (%)	164 (76)	138 (64)	0.006	−2.75
Pulmonary hypertension, *n* (%)	54 (25)	29 (13.5)	0.005	−3.08
Diabetes mellitus, *n* (%)	44 (20)	29 (13.5)	0.07	−1.92
Stroke, *n* (%)	13 (6)	8 (3.7)	0.26	−1.11
Cancer, *n* (%)	13 (6)	9 (4.2)	0.42	−4.36

**Table 2:** Baseline characteristics of patients < 60 years compared to patients > 60 years.

	Pts. < 60 y *n* = 214	Pts. > 60 y *n* = 214	*p*	Standardised Mean Difference
Sex, men	139 (64.9)	150 (70)	0.40	−3.51
Weight, kg	80 ± 14	82 ± 6	0.80	1.92
Height, cm	180 ± 16	179 ± 16	0.98	−2.90
Chronic obstructive pulmonary disease, *n* (%)	11 (5.1)	13 (6)	0.90	−2.96
Peripheral vascular disease, *n* (%)	1 (0.46)	5 (2.3)	0.21	1.49
Hyperlipoproteinemia, *n* (%)	66 (30.8)	87 (40.6)	0.04	−3.40
Arterial hypertension, *n* (%)	145 (67.7)	157 (73)	0.07	−2.02
Pulmonary hypertension, *n* (%)	43 (20)	40 (18.6)	0.89	−2.56
Diabetes mellitus, *n* (%)	31 (14.4)	42 (19.6)	0.08	−3.81
Stroke, *n* (%)	8 (3.7)	13 (6)	0.26	−1.92
Cancer, *n* (%)	8 (3.7)	14 (6.5)	0.40	−1.11

## Data Availability

The data presented in this study are available on request from the corresponding author. The data are not publicly available due to privacy and ethical reason.

## Supplementary Materials

The following are available online at https://www.mdpi.com/article/10.3390/jcm10235554/s1, Figure S1: Single adverse events in group A, Figure S2: Single adverse events in group B.
