# Supplementary material for: Surgical Aortic Valve Replacement—Age-Dependent Choice of Prosthesis Type
Source: J Clin Med. 2021 Nov 26;10(23):5554. doi: 10.3390/jcm10235554 (PMC8658378; doi:10.3390/jcm10235554)
Supplement: Supplementary file 1 [file jcm-10-05554-s001.zip › jcm-1434276-supplementary.pdf]

Journal of
Clinical Medicine

Supplementary Materials

Figure S1 shows single adverse events in group A.

Figure S2 shows single adverse events in group B.

Single adverse events (Reoperation, Stroke, Major Bleeding)

No significant difference was identified regarding the reoperation rates between patients undergoing bioprosthetic or mechanical valve replacement in group A (p = 0.25). The cumulative incidence rates at 5 years were 0% vs. 1.1 ± 0.1% for bioprosthesis and mechanical prosthesis, respectively. At 10 years cumulative incidence rates were 5.1 ± 0.4% vs. 1.1 ± 0.1% for bioprosthesis and mechanical prosthesis, respectively. Also, no significant difference was identified regarding the reoperation rates between patients with bioprosthetic and those with mechanical prostheses in group B (p = 0.52). At 10 years cumulative incidence rates were 0% vs. 3.8 ± 1.8% for bioprosthetic and mechanical replacement, respectively. The first reoperation of a bioprosthesis in this group was performed 11.8 years after the initial AVR. The reasons for reoperations on the patients with mechanical prostheses included low-INR value because of patient's incompliance with consequent fibrotic debris accumulation on the prosthesis, thrombosis, paravalvular leckage because of infective endocarditis and aortic aneurysm.

Stroke rates were similar for patients with bioprosthetic and mechanical valves in both groups (A p = 0.93, B p = 0.68). In group A cumulative incidence rates at 10 years were 2.2 ± 1.5% vs. 0% for bioprosthesis vs. mecahnical prosthesis, respectivey. In group B cumulative incidence rates at 10 years were 2.2 ± 1.5% vs. 5.1 ± 0.4% for patients with bioprostheses vs. mecahnical prostheses, respectivey.

Major bleeding rates showed no significant difference comparing bioprosthetic with mechanical valves in both groups (A p = 0.56, B p = 0.65). In group A cumulative incidence rates for major bleeding at 5 years were 2.2 ± 0.2% and 1.1 ± 0.2% and at 10 years 2.2 ± 0.3% and 2.4 ± 1.7% for bioprosthesis and mechanical prosthesis, respectively. In group B cumulative incidence rates for major bleeding at 10 years were 0% vs. 1.2 ± 0.1% for bioprosthesis and mechanical prosthesis, respectively.

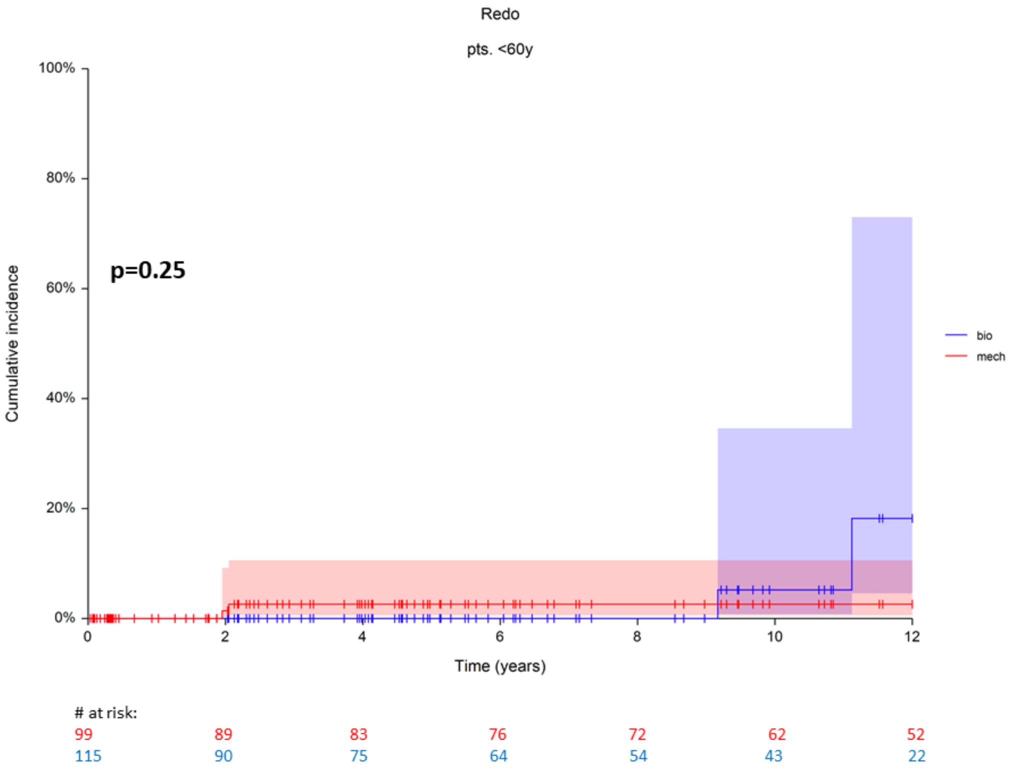

Redo
pts. <60y
p=0.25
Cumulative incidence
100%
80%
60%
40%
20%
0%
0
2
4
6
8
10
12
Time (years)
bio
mech
at risk:
99 89 83 76 72 62 52
115 90 75 64 54 43 22

(**a**)

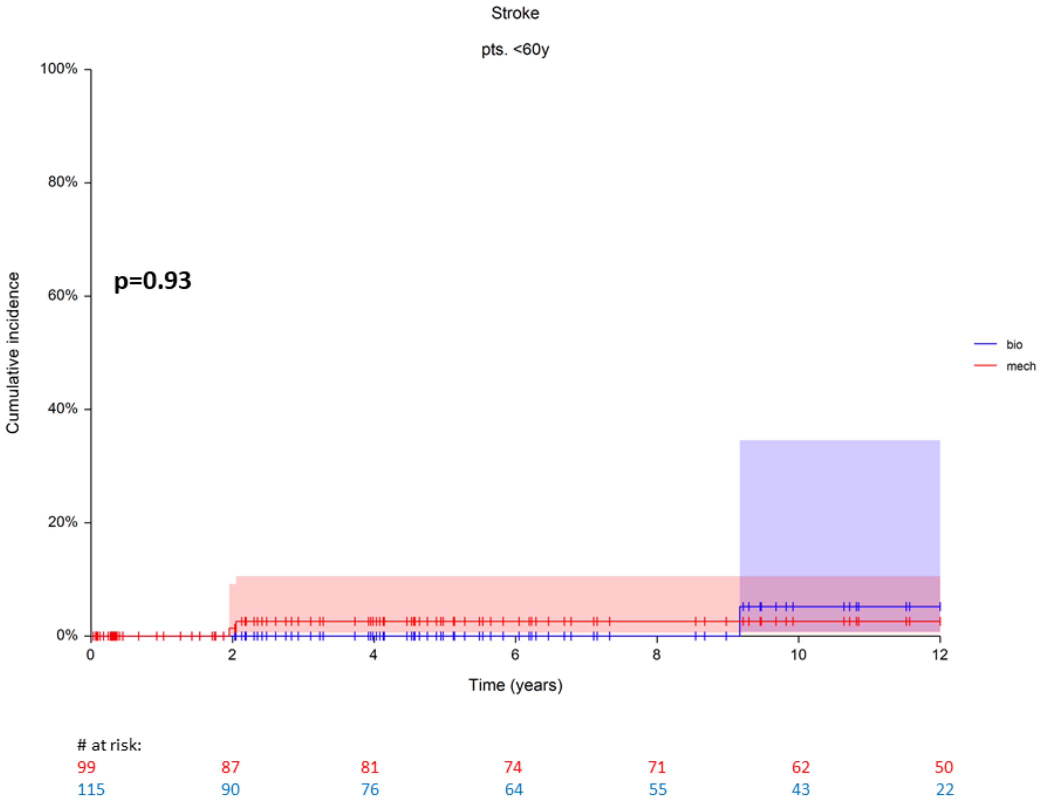

Stroke
pts. <60y
p=0.93
Cumulative incidence
100%
80%
60%
40%
20%
0%
0
2
4
6
8
10
12
Time (years)
bio
mech
at risk:
99 87 81 74 71 62 50
115 90 76 64 55 43 22

(**b**)

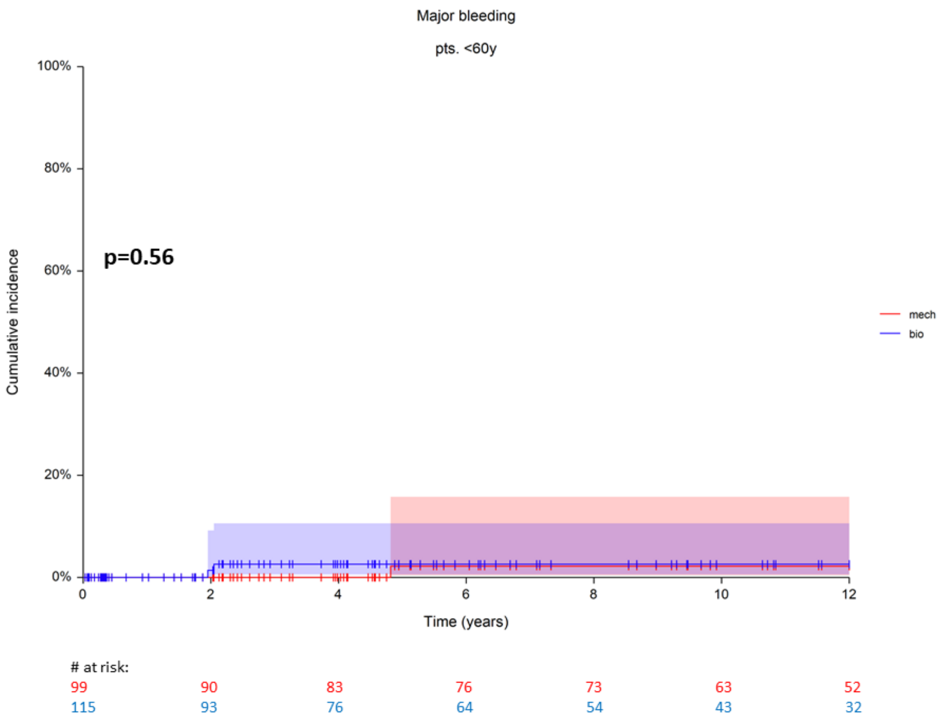

(c)

Figure S1. Single adverse events in group A. **(a).** Cumulative incidence rates for reopearation of the aortic prosthesis in group A. **(b).** Cumulative incudence rates for stroke in group A. **(c).** Cumulative incudence rates for major bleeding in group A. Red numbers are presenting the patients with mechanical prosthesis, blue numbers are presenting the patients with bioprosthesis.

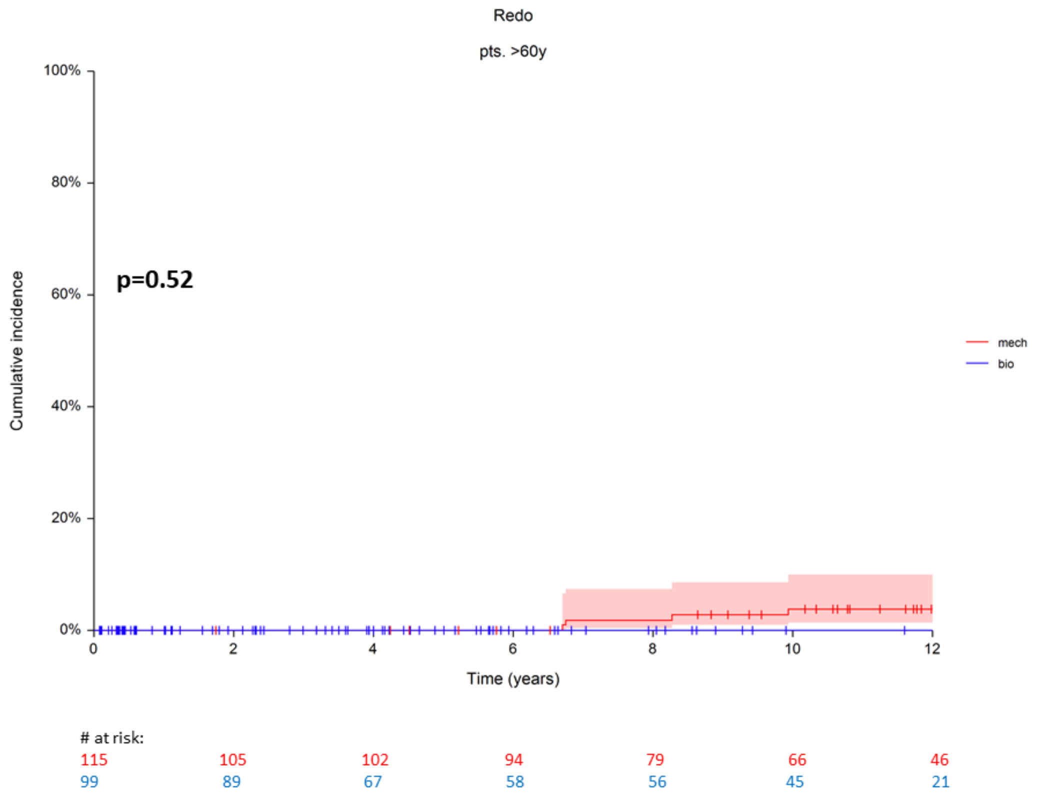
Redo
pts. >60y
p=0.52
Cumulative incidence
100%
80%
60%
40%
20%
0%
0
2
4
6
8
10
12
Time (years)
mech
bio
at risk:
115 105 102 94 79 66 46
99 89 67 58 56 45 21

(**a**)

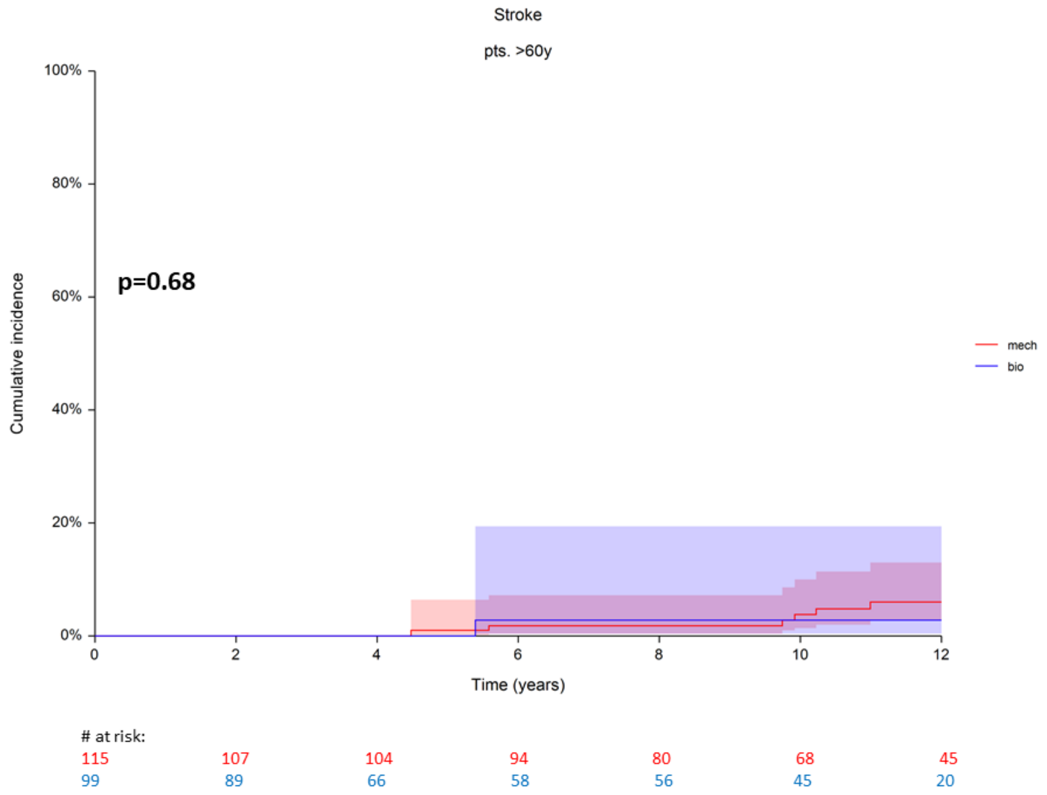
Stroke
pts. >60y
p=0.68
Cumulative incidence
100%
80%
60%
40%
20%
0%
0
2
4
6
8
10
12
Time (years)
mech
bio
at risk:
115 107 104 94 80 68 45
99 89 66 58 56 45 20

(**b**)

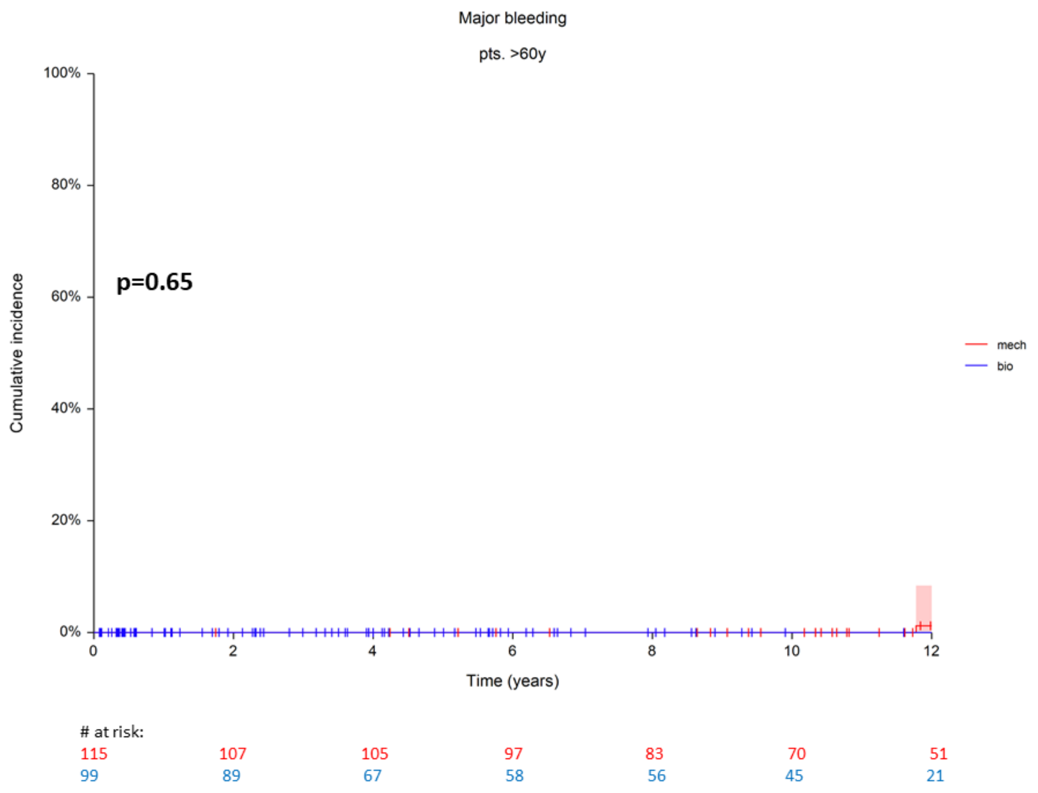

(c)

Figure S2. Single adverse events in group B. **(a)**.Cumulative incidence rates for reopearation of the aortic prosthesis in group B. **(b)**. Cumulative incudence rates for stroke in group B. **(c)**. Cumulative incudence rates for major bleeding in group B. Red numbers are presenting the patients with mechanical prosthesis, blue numbers are presenting the patients with bioprosthesis.
